# Manifestaciones orales del síndrome de Maroteaux-Lamy (Mucopolisacaridosis VI)

**DOI:** 10.21142/2523-2754-0901-2021-051

**Published:** 2021-03-11

**Authors:** Sandra Viviana Cáceres Matta, Luis Eduardo Carmona Arango

**Affiliations:** 1 Grupo de Investigación PROMOUC, Programa de Odontología, Facultad Ciencias de la Salud de la Universidad del Sinú Seccional Cartagena. Cartagena, Colombia. scacerem09@gmail.com Universidad del Sinú Grupo de Investigación PROMOUC, Programa de Odontología Facultad Ciencias de la Salud Universidad del Sinú Seccional Cartagena Cartagena Colombia scacerem09@gmail.com; 2 Grupo de Investigación PROMOUC, Posgrado de Odontopediatría y Ortopedia Maxilar, Facultad de Odontología de la Universidad de Cartagena. Cartagena, Colombia. lcarmonaa@unicartagena.edu.co Universidad de Cartagena Grupo de Investigación PROMOUC, Posgrado de Odontopediatría y Ortopedia Maxilar Facultad de Odontología de la Universidad de Cartagena Cartagena Colombia lcarmonaa@unicartagena.edu.co

**Keywords:** mucopolisacáridos, arilsulfatasa, dermatán sulfato, hiperplasia gingival, mucopolysaccharides, arylsulfatase, dermatan sulfate, gingival hiperplasia

## Abstract

La mucopolisacaridosis tipo VI, también conocida como síndrome de Maroteaux-Lamy, es un trastorno lisosómico autosómico recesivo, causado por la deficiencia de la enzima arilsulfatasa B, lo que conduce a la acumulación de dermatán sulfato en los tejidos y su excreción urinaria. La deposición de mucopolisacáridos genera un trastorno progresivo que afecta a múltiples órganos y que, a menudo, resulta en la muerte a temprana edad. Esta enfermedad tiene varias manifestaciones orales, entre las que destacan las complicaciones dentales, que pueden ser graves e incluir folículos similares a quistes dentígeros, maloclusiones, defectos condilares e hiperplasia gingival, además de características clínicas como cuello corto, opacidad corneal, macroglosia y agrandamiento del cráneo, dimensión anteroposterior larga y mano en garra. Se presenta el caso de un paciente de 14 meses de edad que acudió a consulta de odontopediatría por episodios de fiebre, bajo peso e hiperplasia gingival severa. El examen físico evidenció *facies tosca*, cuello corto, *pectus excavatus*, manos con disminución en agarre y retardo en el neurodesarrollo. El examen intraoral halló retardo de la erupción dental, hiperplasia gingival generalizada y paladar con poco crecimiento transversal. El examen radiográfico detectó órganos dentarios incluidos y mala posición en el sector anterior, molares superiores dentro del seno maxilar y caninos inferiores rotados. El paciente fue remitido a medicina para exámenes bioquímicos y genéticos para definir el diagnóstico. La bioquímica reveló MPS tipo VI, lo que fue confirmado mediante prueba molecular. Las manifestaciones clínicas en este caso corresponden a la forma clínica de progresión rápida reportada en estos pacientes: talla baja, malformaciones esqueléticas y alteraciones a nivel oral. Los niños con MPS VI grave comienzan temprano y progresan rápidamente, las radiografías óseas y la medición de GAG en orina son útiles para el diagnóstico con actividad de la enzima ARSB y genética. Es necesario fortalecer el conocimiento en odontología y la población en general sobre las características clínicas de mucopolisacáridos tipo VI para tener un diagnóstico temprano y un mejor manejo de patologías en estos pacientes.

## Abstract (en)

Mucopolysaccharidosis type VI, also known as Maroteaux-Lamy syndrome, is an autosomal recessive lysosomal disorder, due to the deficiency of the enzyme arylsulfatase B that leads to the accumulation of dermatan sulfate in the tissues and its urinary excretion. Mucopolysaccharide deposition leads to a progressive disorder affecting multiple organs that often results in death at a young age. This disease has several oral manifestations, among which dental complications can be serious and include follicles similar to dentigerous cysts, malocclusions, condylar defects and gingival hyperplasia, in addition to a short neck, corneal opacity, macroglossia, skull enlargement, anteroposterior dimension long, claw hand is some of the clinical features. A case of a 14-month-old patient is presented, who attended a pediatric dentistry consultation for episodes of fever, low weight, severe gingival hyperplasia. Physical examination revealed coarse facies, short neck, pectus excavatus, hands with decreased grip, and neurodevelopmental delay. On intraoral examination, dental eruption delayed, generalized gingival hyperplasia, palate with little transverse growth. On radiographic examination, dental organs included and poor position in the anterior sector, upper molars within the maxillary sinus, rotated lower canines. He is referred to medicine for biochemical tests and genetics for diagnosis. Detailed biochemistry MPS type VI, confirmed by molecular testing. The clinical manifestations in this case correspond to the clinical form of rapid progression reported in these patients. They report: short stature, skeletal malformations and alterations at the oral level. Children with severe MPS VI start early and progress rapidly, bone radiographs and urine GAG measurement are helpful for diagnosis with genetic and ARSB enzyme activity. It is necessary to strengthen the knowledge in dentistry and the general population about the clinical characteristics of type VI mucopolysaccharides in order to have an early diagnosis and management of pathologies in these patients.

## INTRODUCCIÓN

Las mucopolisacaridosis son un grupo de trastornos genéticos que implican alteraciones en el metabolismo de los mucopolisacáridos. Las manifestaciones clínicas son resultado de la acumulación de mucopolisacáridos (glicosaminoglicanos) en varios órganos. El depósito de mucopolisacáridos conduce a retraso en el neurodesarrollo desde los primeros meses de vida. Las mucopolisacaridosis son autosómicas recesivas, con excepción del síndrome de Hunter, que se hereda como un rasgo recesivo ligado al cromosoma X ^1^^,^^2^^,^^3^. 

El síndrome de Maroteaux-Lamy (mucopolisacaridosis tipo VI), descrito por primera vez en 1965 ^4^, se debe a una deficiencia de la enzima arilsulfatasa B (N-acetilgalactosamina4-sulfatasa), que genera la acumulación del dermatán sulfato dentro de los lisosomas ^5^. Normalmente, los pacientes con mucopolisacaridosis tipo VI se diagnostican en los primeros años de vida y las afecciones más las fallas multisistémicas, por lo general, ocasionan la muerte temprana. 

Los pacientes que sufren este síndrome tienen características similares a los que padecen otras mucopolisacaridosis, pero se distinguen por la inclusión de metacromáticas en los leucocitos y una actividad deficiente de la arilsulfatasa B ^6^. Los rasgos característicos de la enfermedad incluyen retraso en el crecimiento, hernias, *facies tosca*, cuello corto, anomalías en la columna, mano en garra y hepatoesplenomegalia (resultado del depósito de mucopolisacáridos en varios órganos). En la mayoría de los casos, la muerte se debe a la infección del tracto respiratorio o a una enfermedad cardíaca. 

Las características creneofaciales en este síndrome son similares a las del síndrome de Hurler (MPS tipo I). La cabeza presenta un aumento de tamaño, con frente prominente, a menudo la zona supraorbital; crestas y protuberancias temporales marcadas. La rinitis con rinorrea y la opacidad corneal son comunes en estos pacientes. A nivel de cavidad oral, se observa incompatibilidad labial y macroglosia. Algunas investigaciones han reportado órganos dentarios en forme de clavija, anormales, con anomalías de número y forma, al igual que defectos en la calcificación dental como hipoplasias; normalmente, existe una mordida abierta anterior asociada a la macroglosia ^7^^,^^8^. 

El objetivo de este artículo es describir las características clínicas odontológicas específicas de un paciente portador del síndrome de Maroteaux-Lamy (mucopolisacaridosis tipo VI).

## REPORTE DE CASO

Una niña de 14 meses de edad, de procedencia rural, del departamento de Bolívar (Colombia), con síndrome de Maroteaux-Lamy (mucopolisacaridosis tipo VI), fue remitida al Posgrado de Odontopediatría y Ortopedia Maxilar de la Universidad de Cartagena (Colombia) para el diagnóstico odontológico como complemento del reporte médico, el cual señalaba hepatoesplenomegalia leve. La excreción urinaria de mucopolisacaridosis media, con la prueba de azul de dimetilmetileno, desde el área de bioquímica, arrojó 54,7 mg/mmol de creatinina (valores normales: <12,2 mg/mmol creatinina). La electroforesis bidimensional de mucopolisacaridosis en muestra de orina mostró un gran aumento de dermatán sulfato, lo que sugiere mucopolisacaridosis tipo VI. La deficiencia enzimática de la arilsulfatasa B fue identificada por ensayo de enzima ASB ^9^ de los fibroblastos cultivados de la paciente, la actividad de ABS fue 120 y el rango control de 300 a 900 nmol/h/mg. 

**Figura 1:**
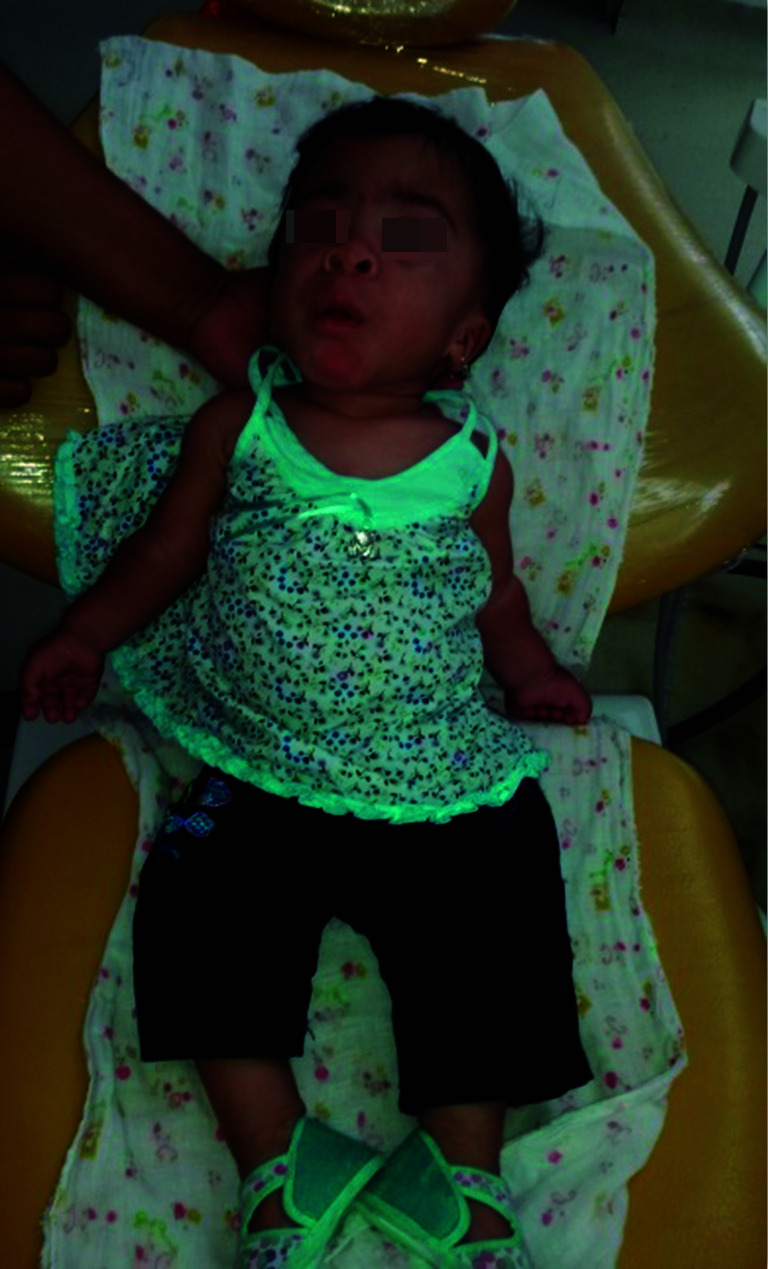
Esta fotografía muestra la facies característica del síndrome de Maroteaux-Lamy.

**Figura 2A:**
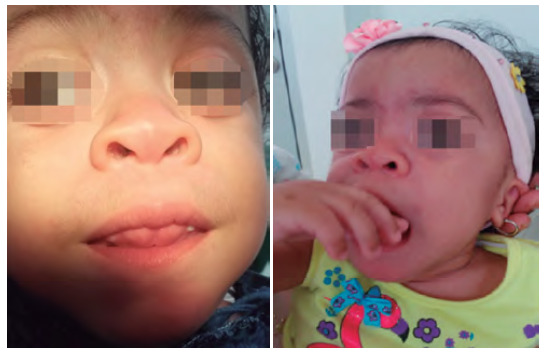
y 2B. Cabeza grande, cuello corto, incompetencia labial por hiperplasia gingival y leve lengua agrandada, agrandamiento del cráneo y una dimensión anteroposterior larga son características típicas del síndrome de Maroteaux-Lamy.

De igual manera, la paciente en la historia clínica médica reportaba insuficiencia cardiaca y respiratoria. Los exámenes radiográficos evidenciaron rasgos típicos como disostosis múltiple. El consentimiento informado escrito y verbal se obtuvo antes de la documentación del caso; asimismo, se le explicó a la madre de la paciente cada uno de los procedimientos odontológicos por desarrollar. Los hallazgos extraorales e intraorales de la paciente también fueron característicos del síndrome Maroteaux-Lamy, pues presentaba retraso en el desarrollo psicomotor, cuello corto, mano en forma de garra (Figura 1), mordida abierta anterior asociada con hiperplasia gingival y leve macroglosia, agrandamiento leve del cráneo y una dimensión anteroposterior aumentada, puente nasal aplanado e hipertelorismo, cejas pobladas, las pestañas estaban presentes (Figuras 2A y 2B).

**Figura 3A y 3B:**
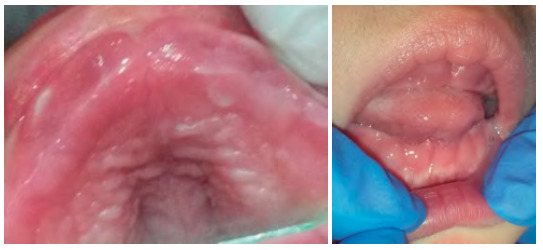
Hiperplasia gingival generalizada en maxilar superior e inferior, retraso en la erupción de los dientes anteriores, paladar profundo con poco desarrollo en el crecimiento del plano medio sagital, macroglosia.

El examen clínico intraoral reveló hiperplasia gingival, retraso en la erupción dental de órganos dentarios anteriores, agradamiento de procesos alveolares, paladar profundo, estrecho en sentido sagital y trasversal, macroglosia (Figuras 3A y 3B). 

**Figura 4:**
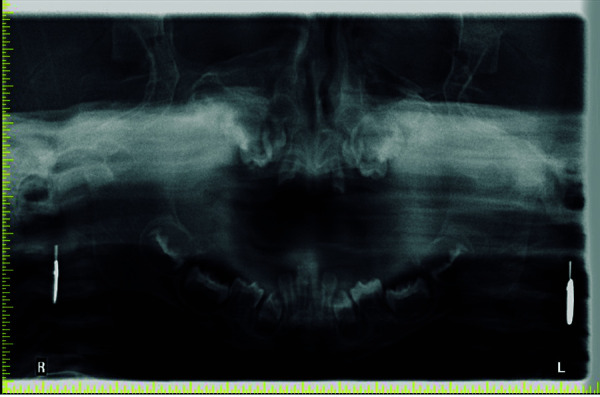
La radiografía panorámica muestra los dientes no erupcionados y varias radiolucencias pericoronales.

**Figura 5:**
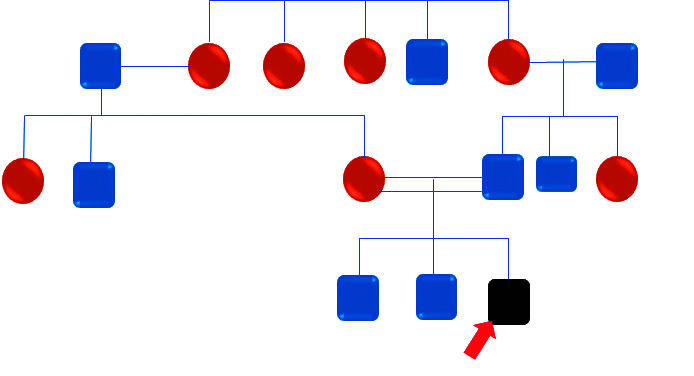
El familiograma del árbol genealógico de tres generaciones de la familia reveló consanguinidad.

Se tomó radiografía panorámica en circunstancias difíciles (la paciente no cooperaba y estaba ansiosa), el estudio radiográfico reveló dientes no erupcionados con radiolucencias pericoronales que se asemejaban a quistes dentígeros (Figura 4). El árbol genealógico de 3 generaciones de la familia reveló consanguinidad (Figura 5). 

## DISCUSIÓN

Este raro síndrome es una enfermedad autosómica recesiva causada por mutaciones en la enzima arilsulfatasa B y el gen ARSB, necesario para la degradación de dermatán sulfato. Un defecto en el gen da como resultado la acumulación de mucopolisacáridos no degradados o parcialmente degradados, que luego interfieren con la función de las células afectadas, lo que da como resultado labios grandes, gruesos, hiperplasias gingivales y opacidades corneales ^10^^,^^11^. Su prevalencia es baja y varía de 1:100 000 nacidos a 1:1300 000, en diversas poblaciones ^12^. La acumulación progresiva de mucopolisacáridos en tejido blando causa complicaciones como insuficiencia cardiaca y bronconeumonía, que ocasionan la muerte de estos pacientes en los primeros años de vida ^7^. 

Diversas investigaciones describen a los órganos dentarios de estos pacientes como de tamaño pequeño y espaciados, de igual manera, y se han reportado anomalías en número y forma con alteraciones en la calcificación, mordida abierta anterior asociada con la hiperplasia gingival y macroglosia ^13^. Los rasgos faciales característicos de Maroteaux-Lamy son un aumento de tamaño de la cabeza en la zona anteroposterior y cuello corto (figura 1). Aunque haya erupción tardía de incisivos, se evidencia hipocalcificación con alteración de forma. Kantaputra reportó que los pacientes con síndrome de Maroteaux-Lamy tienen muy poca caries dental. Las hiperplasias gingivales y la hipertrofia de la cresta alveolar maxilar ^5^ se reportan como las principales manifestaciones orales del síndrome, tal como se evidencia en las figuras 3A y 3B. 

Además de estos hallazgos orofaciales, el síndrome también describe áreas radiolúcidas que se asemejan a folículos similares a un quiste dentígero ^13^. Se detectaron áreas radiolúcidas similares en nuestra paciente (figura 4); los márgenes de la radiolucencias suelen ser suaves y claramente definidos. Estas radiotransparencias son causadas por la acumulación de mucopolisacáridos en tejidos ^1^^,^^2^. El odontopediatra tiene un papel relevante en el cuidado de la salud bucal de estos pacientes. 

El estudio de las enfermedades de almacenamiento de carbohidratos es sumamente importante para comprender cuáles son las consecuencias que puede tener un defecto genético que se expresa en el mal funcionamiento de un organelo, ya que debido a su poca frecuencia en la población no se tiene presente su comportamiento y resulta difícil crear un tratamiento efectivo. Se debe tener en cuenta que la calidad de vida de las personas que las padecen es muy baja y por ello es necesario ampliar los estudios, pues por lo general solo reciben cuidados de naturaleza paliativa. La aplicación de métodos de vanguardia en diversos campos para unificarlos podrían hacer realidad un tratamiento efectivo, enfocado en la edición genética, de forma que se pueda combatir de raíz las enfermedades de carácter autosómico recesivo, como la mucopolisacaridosis, y de esta forma tener certeza de obtener un resultado con una efectividad muy alta y tal vez definitiva ^3^^,^^4^.

De acuerdo con lo anterior, las enfermedades lisosomales en conjunto tienen una incidencia de 1:7700 nacidos vivos, cantidad a considerar por el gremio odontológico que, como en el presente caso, pueden ser los llamados a realizar un diagnóstico temprano, basado en la cantidad de manifestaciones orales presentes, que convierten al profesional de la odontología en una remisión obligada. Al revisar las manifestaciones clínicas generales de las enfermedades lisosomales ^13^, solo la macroglosia es reportada como un hallazgo frecuente, patología presente en muchos síndromes. Se hace necesario que el odontólogo y, específicamente, el odontopediatra conozca casos clínicos donde se presenten otros hallazgos que le permitan relacionar las manifestaciones generales con las intraorales, y brindar así una orientación y un manejo efectivo de estas patologías huérfanas.

## CONCLUSIONES

El pronóstico de estos pacientes varía en función de la edad de aparición, de la velocidad de progresión de la enfermedad, de la edad de inicio del tratamiento del suplemento enzimático y del manejo interdisciplinario. Esta patología se caracteriza por ser crónica y progresiva, por lo que el inicio temprano del tratamiento permite mayores logros terapéuticos y mejor calidad de vida para los pacientes y sus familias. Por ello, es necesario que exista mayor conocimiento entre la comunidad médico-odontológica y la población en general sobre las características clínicas de la mucopolisacaridosis tipo VI, para lograr un diagnóstico temprano y un mejor manejo de las patologías que presentan estos pacientes portadores del síndrome.

## Fuente de financiamiento:

Autofinanciado
